# Hope and Fear of Threats as Predictors of Coping with Two Major Adversities, the COVID-19 Pandemic and an Armed Conflict

**DOI:** 10.3390/ijerph19031123

**Published:** 2022-01-20

**Authors:** Hadas Marciano, Yohanan Eshel, Shaul Kimhi, Bruria Adini

**Affiliations:** 1 Stress and Resilience Research Center, Tel-Hai College, Tel Hai 1220800, Israel; yeshel@psy.haifa.ac.il (Y.E.); shaulkim@telhai.ac.il (S.K.); 2 The Institute of Information Processing and Decision Making (IIPDM), University of Haifa, Haifa 3498838, Israel; 3 Department of Psychology, University of Haifa, Haifa 3498838, Israel; 4 Department of Emergency and Disaster Management, School of Public Health, Sackler Faculty of Medicine, Tel Aviv University, Tel Aviv 6139001, Israel; adini@tauex.tau.ac.il

**Keywords:** COVID-19, hope, resilience, anxiety, depression, distress, perceived threats, armed conflict

## Abstract

Coping with adversities has been explained by two major theories: the fear appeal theory and the hope theory. The predictability of hope with that of fear of threats as variables explaining coping with two major adversities, the COVID-19 pandemic and an armed conflict, was compared. Participants were approached via an internet panel company in two different times: (1) January 2021 (*N* = 699; age range: 18–82; 330 women), during the third wave of the COVID-19 pandemic in Israel and (2) May 2021 (*N* = 647; age range: 19–83; 297 women), during an armed conflict between Israel and Hamas. Participants self-reported on hope, four perceived threats (health, economics, security, and political), well-being, individual resilience, societal resilience, and distress symptoms (anxiety and depression symptoms) were collected. Hope was found as a more consistent and stronger predictor of the following expressions of coping: well-being, individual and societal resilience, depression, and anxiety. It can be concluded that hope is a better and more consistent predictor of coping, as well as coping suppressing expressions, compared with fear of threats, in the face of the current adversities. The innovative nature of these findings, the importance of hope as a coping supporter, and the need for replicating these innovative results are discussed and elaborated.

## 1. Introduction

Studies found that positive as well as negative emotions occur throughout intensely stressful periods [1,2,3]. This duality of emotional responses raises the question of whether coping with adversity is determined mainly by positive emotions like hope, or by negative emotions like fear. 

One perspective claims that the effectiveness of coping reflects the level of threats caused by the menacing events [4]. A critical factor in understanding a population’s response to a threat is the fear it elicits. Fear is an important predictor of behavioral changes and health-securing behaviors, as suggested by the fear appeal theory [5,6]. It has been suggested that the rapid spread of the COVID-19 virus, its high lethality, and the lack of pharmaceutical prevention or cure, have negatively affected coping with this global pandemic [4]. 

A different perspective claims that coping with major crises is mainly determined by the level of individual’s hope rather than by fear of its perceived threats. In this “hope theory” perspective, hope was defined as “a positive motivational state that is based on an interactively derived sense of successful of (a) agency (goal-directed energy) and of (b) pathways planning to meet goals” [7] (p. 287). This view sees hope as a twofold positive expectation about one’s agency (capability to obtain the personal ambition), and one’s pathways (capability to identify means and strategies to get the desired goals). Others concluded that hope is particularly suited for explaining and promoting positive coping with adversities [8]. Hope is conceived of as a form of self-confidence and sense of personal mastery in the service of goal pursuit, planning, and problem solving [9]. Consequently, it is associated with the personal characteristics of individual [10], perceived competency and actual achievement in various domains [11], as well as feelings of self-worth and self-esteem [12]. Furthermore, hope depends to a great extent on such attitudes as persistence, patience, and readiness to take advantage of favoring conditions [13].

Earlier psychodynamic theoreticians have described hope as an important factor in psychotherapy. For example, both Freud and Menninger claimed that often psychotherapeutic gains may be explained by increased hope throughout the process of treatment [14,15]. Others perceived hope as crucial for the success of any psychotherapy [16,17]. The Holocaust survivor, psychiatrist Viktor Frankl, also expressed similar ideas about the importance of hope, in the form of “sense of meaning” [18]. Similarly, we also believe that hope is an essential antecedent of coping with stress, which is constantly employed by most individuals throughout their lives and helps them to overcome many frights they may encounter.

It was found that hopeful individuals enjoy many benefits, which are not experienced by their low-hope counterparts, including superior academic achievement, psychological adjustment, and physical health [19]. The major role of hope in the face of adversity is presented in an essay claiming that coping and hope are mutually dependent, as hope underlies all coping efforts [20]. According to this model when the probability of a good outcome is higher, hope facilitates coping, whereas with a lower probability of a good outcome, people increase hope and reduce threats by reappraising and improving their perceived personal odds. Individuals with high hope levels are expected to appraise stressors as more challenging than threatening, and consequently are more motivated to find solutions to ameliorate the stressful feelings. Using such appraisals was found as facilitating coping with a serious life-threatening disease [21].

The available research seems to support the major role of hope in coping with adversities. Hope has been associated with the use of adaptive coping methods [22], more positive appraisals of stressful events [23] and more flexible thoughts during a crisis [24]. A study on childhood cancer survivors found that hope was positively associated with posttraumatic growth and negatively associated with depression and anxiety [25]. According to [26], hope is the most important in unpredictable and uncontrollable circumstances, such as minority adolescents living in urban environments. Indeed, research has found that ethnic minority individuals with higher sense of hope, tend to employ more problem-solving coping strategies and fewer avoidant coping approaches [27], have greater coping efficacy [28] and higher academic performance [29].

A comprehensive analysis of hope and fear claims that hope is distinguished from other connected attitudes such as optimism, trust, and faith. Hope is similar to positive expectations, because it implies a wish or goal that the anticipated event will materialize. The basic cognitive ingredients of hope are: (a) the belief that an event is possible, (b) the goal or wish that this event will be obtained, and (c) the belief that the event’s achievement is not completely within one’s control. It was further suggested that the cognitive ingredients of fear, as in hope, are wish and belief of possibility. For, being afraid about an event, it is sufficient to believe that this event is possible, and to have an opposite wish or goal that the event will not occur [13].

Adversities tend to increase individual stress, which is a response to a situation that is appraised by the individual as personally significant and as exceeding one’s available coping resources [30]. It was found that positive as well as negative emotions occur throughout intensely stressful periods [1,2,3]. Coping constitutes the thoughts and behaviors people use to manage the internal and external demands of stressful events.

In the current study, we explore hope and fear of threats as predictors of several psychological responses, in face of two different adversities: the COVID-19 pandemic and an armed conflict. A recent review suggests that the fears and anxieties raised by natural disasters, past epidemics and, more specifically, the COVID-19 pandemic, often result in numerous immediate and long-term emotional distress effects, such as uncertainty, distrust, and post-traumatic stress disorder (PTSD) [31]. An additional world-wide review indicates that fears of threats of the COVID-19 pandemic are associated with relatively high rates of depression, anxiety, PTSD, and psychological distress [32]. According to this literature review, fears of threats have tremendous negative impacts on people’s mental health, including worries, sleeping disorders, concentration difficulties, and increased use of poor coping mechanisms. Similarly, a comprehensive review of the psychological effects of armed conflicts shows that these adversities also tend to result in decreased coping abilities and higher levels of depression, anxiety, and PTSD [33]. A study of the psychological costs of the Vietnam War indicated that these costs extended beyond PTSD and depression, including an increased risk of personality disorders, suicide, and alcohol abuse [34]. 

Pandemics and armed conflicts constitute risks for the general public in light of the possible damage that they may bring about [35,36]. Examination of the COVID-19 pandemic perceived risks shows that its major health threats are often accompanied by secondary threats [37]. A study carried out in the USA, the UK, and Israel, showed that individuals in all three countries experienced economic anxiety, which was equal to the health anxiety, and was higher than the routine-change and isolation anxiety [38]. 

Note that Israel is constantly exposed to a large number of long-lasting dangers. It was claimed that the Israeli society is continually threatened by a surplus of risks and challenges: geopolitical and foreign policy challenges, borders and settlements issues, regional security threats, and social and economic contexts [39]. Repeated rounds of armed conflicts between Israel and the Palestinians in the Gaza Strip have erupted in the past decades [40]. Most of these conflicts occur for a relatively short duration, lasting for a few days to a few weeks [41]. The Israeli public is familiar with various kinds of limited armed conflicts and generally perceive that they are protected from harm, due to protective measures that are available, mostly the Iron Dome (which intercepts missiles). The COVID-19 pandemic, in contrast, constitutes an unfamiliar emerging threat that has occurred lately for the first time and which involves unknown immediate consequences and future impacts; moreover, there is no way to determine whether and when it will be contained. Furthermore, the number of Israeli confirmed cases of the pandemic, as well as the overall mortality and morbidity was much higher, compared with the victims of the last armed conflicts. Consequently, the level of uncertainty due to the COVID-19 pandemic is much higher, compared with the armed conflicts that Israel has been involved with in the past decades [42]. In this context, we assume that a sense of hope is more crucial in coping with the COVID-19 pandemic than with the ongoing security issues. 

### 1.1. Psychological Coping Indicators 

Four different coping indicators were chosen to measure the participant’s coping with the two adversities. All indicators are well-established measurements in the literature on stress and resilience, as described below: 

**Well-being:** Well-being was defined as the combination of feeling good and functioning well; the experience of positive emotions (happiness and contentment), the development of one’s potential, having control over one’s life, having a sense of purpose, and experiencing positive relationships [43]. High positive correlations were found between well-being, happiness, psychological quality of life, life satisfaction, positive effects, and individual resilience [44]. Well-being was negatively correlated with neuroticism and psychological inflexibility, and positively correlated with extraversion and interpersonal contact [45].

**Individual resilience:** Individual resilience is “a positive adaptation within the context of significant adversity” [46] (p. 543). It constitutes a stable trajectory of healthy functioning after a highly adverse event [47]. Individual resilience was described as a major factor in reducing the negative impacts of traumatic events [48]. Research shows positive correlations between individual resilience and measures of well-being and hope, and negative correlations between individual resilience and distress symptoms, as well as a sense of danger [49]. 

**Societal resilience:** (which was formerly termed “national resilience”): Societal resilience reflects a successful societal adjustment and efficient functioning following potentially traumatic events [50]. Societal resilience was defined as the nation’s ability to successfully cope with its adversities (e.g., poverty, terrorism, or corruption) while keeping its social fabric intact [51]. Societal resilience was negatively correlated with distress symptoms and with a sense of danger and was positively correlated with a sense of coherence [52].

**Psychological coping suppressing factors (distress):** The COVID-19 outbreak has been associated with psychological distress responses of grief, hopelessness, posttraumatic symptoms, panic attacks, anxiety, depression, loneliness, ambivalence, fear, and concern towards socioeconomic status [53]. A recent review found a high prevalence of distress among the investigated general populations throughout the COVID-19 pandemic [54]. These distress responses have been negatively correlated with a sense of well-being and with individual, community, and societal resilience [55].

### 1.2. Current Study

There is limited research on the relative impacts of fear of threats during major crises in comparison to the effects of hope for a successful recovery, on measures of coping with stressful conditions. Furthermore, a theoretical analysis of the interaction between fear and hope has yet to achieve a clear-cut conclusion. According to [56], fear is a primary emotion that reflects the experienced present and the memorized past, whereas hope is a secondary emotion based on complex processes of creativity and flexibility. Consequently, this perspective claims that fear generally dominates hope, since hope is often preceded and inhibited by the spontaneous, automatically activated, and more encompassing emotional responses of fear. In contrast, these authors also argued that people are not condemned to be ruled by fear. Rather, they can overcome it by using determination, and by establishing “hope orientation” which facilitates change in situations dominated by fear. Recent longitudinal research [57] has found that active coping strategies (personal hygiene practice, support seeking, and positive reappraisal) at T1 were positively associated with T2 hope.

The present study investigates the relative impacts of hope and four perceived threats (health, economic, security, and political threats, which are viewed as sources of fear) on coping indicators during two different adversities: the COVID-19 pandemic and armed conflict. Adversities that are perceived by individuals as more threatening require these individuals to invest greater efforts in coping with them. Successful psychological coping with greater hardships requires a higher level of hope [58]. Furthermore, there is reason to believe that more difficult coping requirements benefit most from hope. The more difficult cases of coping need greater levels of hope and are affected by it to a greater extent. Therefore, it is assumed that hope will predict the three indicators of coping and the two markers of resilience suppression better than the threats of potential risks. It stands to reason that hope expressed by people will represent their actual feeling better than their reports concerning their fears. 

We assume that crisis raises fears as well as hope. In the service of goal pursuit, planning and problem solving, hope is associated with self-confidence and sense of personal mastery, individual agency [10], perceived competency [11], as well as persistence [13]. It is hypothesized, therefore, that:

- Individuals’ hope level will positively predict the positive coping responses (expressed by individual resilience, societal resilience, and well-being), and will negatively predict the sense of distress (expressed by anxiety and depression symptoms). In contrast, individual’s level of fear of potential threats will negatively predict the positive coping responses and will positively predict the sense of distress.
- In line with this central role of hope in supporting coping [20,24], and due to different personal meanings of the potential threats for different individuals, we hypothesize that hope will be a better and more consistent predictor of coping and coping suppressing responses, compared with the investigated perceived threats.
- The effects of hope will be replicated in coping with the two studied adversities: the COVID-19 pandemic and the May 2021 armed conflict between Israel and the Gaza Strip.

## 2. Materials and Methods

### 2.1. Participants 

The data were collected via an internet panel company possessing a database of above 65,000 residents from all demographic sectors and geographic locations of Israel (https://sekernet.co.il/) (accessed on 24 May 2021). A stratified sampling method was employed, aligned with the data of the Israeli Central Bureau of Statistics, appropriately representing the varied groups of the Israeli Jews population (regarding gender, age, and geographic dispersal). The questionnaire was approved by the Ethics Committee of Tel Aviv University and all the participants signed a consent form. 

The present samples are parts of a longitudinal study. The data was gathered in two different circumstances: (1) 14–18 January 2021, during the third wave of the COVID-19 pandemic in Israel, and (2) 22–24 May 2021, during the hostility between Israel and the Hamas in Gaza Strip. Table 1 presenting the demographic characteristics of the investigated samples indicates the similarity of these two samples. The first sample (699 participants, 330 women), ranged between 18 and 82 years of age; 56% of them reported income below the national average; 50% of them held right-wing political attitudes; 50% were secular; 28% were traditional. The second sample (647 participants, 297 women; 593 of them were derived from the first sample), ranged between 19–83 years; 53% of them reported income below the national average; 52% held right-wing political attitudes; 51% were secular; 27% were traditional. Importantly, these characteristics are representative of these demographic features in the general Israeli population. 

### 2.2. Health and Security Situation

The COVID-19 pandemic started in Israel in February 2020. Until May 2021 (the second sampling in the current study), the pandemic continued in three main waves and substantially receded at the beginning of 2021, following a successful vaccination campaign. By 19 April 2021, 88% of individuals above the age of 50 were vaccinated with two doses [59]. The number of new COVID-19 patients has significantly receded on April–May 2021 [60]. Thus, during the first sampling of the current study, the plague was at one of its peaks, while, during the second sampling, it was at a much lower level, as it was (mistakenly) seen like the pandemic faded due to the vaccination campaign. 

In contrast, during the second sampling, the security situation, as well as the socio-political conditions, were quite low. The socio-political conditions before May 2021 were characterized by a general lack of confidence among most of the public in the political leadership and state institutions, which was expressed by major political protests. From the security perspective, the armed clash between Israel and Hamas in the Gaza Strip erupted on 10 May 2021 and lasted for 11 days (10–21 May 2021). It was characterized by massive rocket attacks against civilian communities in the central and southern parts of Israel. A few rocket attacks were also aimed at Northern Israel, from Lebanon. In addition, domestic violent riots between Arabs and Jews also spread in many areas of Israel.

### 2.3. Tools

#### 2.3.1. Predictors

**Hope:** The present scale is based on an earlier scale designed to measure the level of hope for peace between Israel, the Arab nations, and the Palestinians [56,61]. Its two dimensions are personal and collective hope. The current scale of hope included five items, two refer to the personal level (e.g., “I hope that I will emerge strengthened from the crisis”), and three refer to the collective level (e.g., “I hope that the Israeli society will emerge strengthened from the crisis”). The response scale ranged from 1 = very little hope to 5 = high hope. The internal reliability of the scale in the present study was high, α = 0.93 in both samples.

**Perceived threats:** Each of the four investigated perceived threats was determined by a single item which was phrased similarly with a difference in the specific threat wording: “How much do you feel threatened these days by the (health/ economic/security/political) risk?”. The 5-point response scales ranged from 1 = not threatening at all to 5 = threatening very much. Note that each of these four possible threats can bring about a different harm to the individual. To feel fear about something is to believe that it may happen, leading to a wish or strive that it will not materialize [13]. Therefore, we believe that the need to assess the personal sense of each threat, can provide a good estimation of fear of those threats.

#### 2.3.2. Predicted Variables

**Well-being:** The scale consisted of nine items concerning individuals’ perception of their present lives in various contexts, such as work, family life, health, and others. Responses ranged from 1 = very bad to 6 = very good [49,55]. The scale reliability in the present study was high (COVID-19 sample: α = 0.87; Armed conflict sample: α = 0.88).

**Individual resilience:** The individual resilience was measured by the 10-item Connor–Davidson scale (CD-RISC 10) [62,63], portraying individual feelings of ability and power in face of difficulties (e.g., “I manage to adapt to the changes”). Responses ranged from 1 = not true at all to 5 = generally true. The reliability of the scale in the present study was high (α = 0.91 in both samples).

**Societal resilience:** The Societal (ex-National) Resilience Scale was employed [52]. This 13-item tool pertained to trust in national leadership, patriotism, and trust in major national institutions (e.g., “I love my country and am proud of it”). In the current study, three items regarding the specific adversity were added (e.g., “I have full faith in the ability of my country’s health system to care for the population in the current crisis”). The 6-point response scale ranged from 1 = very strongly disagree to 6 = very strongly agree. The reliability of this scale in the present study was high (COVID-19 sample: α = 0.92; Armed conflict sample: α = 0.90).

**Distress:** The sub-scales of anxiety and depression, derived from the Brief Symptom Inventory were employed (BSI) [64]. The five items of the depression subscale pertain to a bad mood, loneliness, lack of interest in anything, feelings of worthlessness, and hopelessness. The four items anxiety subscale refer to felt nervousness, tension, and restlessness. Each item was rated on a scale ranging from 1 = not suffering at all to 5 = suffering very much. Reliabilities for both subscales were high (α = 0.90 in both samples).

## 3. Results

**Figure 1:**
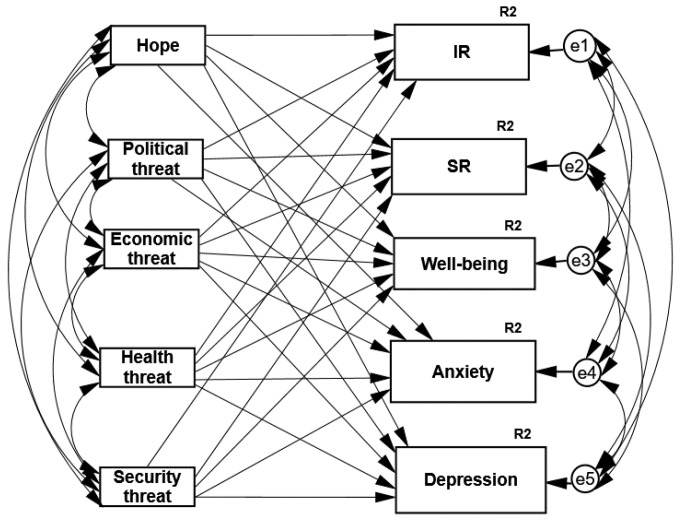
The general theoretical model of the two path analyses—hope and four types of threats predicting individual, societal resilience, well-being, anxiety, and depressive symptoms. IR = individual resilience, SR = societal resilience, R^2^ = explained variance.

Two path analyses of structural equation modeling were utilized with the aid of AMOS software (IBM, SPSS version 26) [65]. We used maximum likelihood estimates and examined a saturated model, since no studies that supported an alternative model were found. Note that, in a saturated model, there is no need to examine model fit, as the default and the saturated model are the same [66]. Standardized scores were employed in these path analyses. We repeated the analysis of the routes twice, one analysis for each one of our two different samples. The two saturated models (all paths were examined) included five predictors: level of hope and the fears of the four perceived risks (political, security, health, and economic), and five predicted psychological coping responses: individual and societal resilience, well-being, anxiety, and depression hypotheses (the general theoretical model can be seen in Figure 1).

As can be seen in the path analyses findings in Table 2, hope consistently significantly, and positively predicted the three direct expressions of coping (individual resilience, societal resilience, and well-being) in both the COVID-19 pandemic and armed conflict measurements. In addition, hope predicted negatively and significantly the two coping suppressing responses (depression and anxiety) in both measurements. In contrast, the four threats predicted these coping indicators less strongly and more sporadically. Thus, hypotheses a and b were supported.

An examination of Table 2 shows that the economic threat significantly predicted all of the five predicted variables in coping with the COVID-19 pandemic but only three in the armed conflict sampling; the political threat significantly predicted three predicted variables in the armed conflict but only two in the COVID-19 sampling; the security threat significantly predicted four of the predicted variables in the COVID-19 sampling and three of the predicted variables in the armed conflict sampling; the health threat significantly predicted all of the five predictors in the COVID-19 pandemic, and four of them in armed conflict. Thus, Table 2 generally supports hypothesis c as well: these effects have been generally replicated in coping with the two different adversities explored.

## 4. Discussion

Two theoretical positions examine factors that explain the development of coping responses throughout two substantial adversities. The first (‘Fear’) claims that the strength of coping negatively reflects the level of threats raised by threatening events [4,6]. The second (‘Hope’) posits that coping with calamities is mainly determined by the level of individuals’ hope [20,26].

‘Fear’ refers to a variety of risk perceptions, beyond the specific jeopardy’s major source of risk, which deal with people’s intuitive evaluations of hazards, including undesirable effects that associate with the main threat. People’s interpretations of the current adversity are influenced by numerous individual, contextual, cultural and social factors [67] that are likely to reduce the correlations between fears of specific threats and coping responses, as indeed suggested by the current findings.

‘Hope’ is based on the positive motivational state of hope, which enables individuals to persevere towards desired goals and pathways that may improve their position [7]. A higher level of hope is expected to affect people’s psychological adjustment by influencing both their appraisal of, and their coping with, the stressors confronted by them. People who possess high hope are also more likely to find benefits in the face of ongoing stressors [23], are less anxious [68], and are more likely to use active coping strategies, such as generating and employing alternative strategies [26].

The current results support each of the above two perspectives. Perceived threats (health, security, economic, and political), as well as the hope level, predicted the investigated indicators of coping with stress: individual and societal resiliencies, well-being, anxiety, and depression. The data also show that, under the investigated circumstances, hope was the most dependable and better predicted all the explored coping indicators. In contrast, fear of threats constitutes a less consistent and less powerful predictor of these coping responses. These results are further supported by being generally replicated in coping with two adversities of different natures: the COVID-19 pandemic and armed conflict. The finding that hope dominate fear in both of these adversities is important because it shows that people use hope as a meaningful means of coping with different situations that may put them and their loved ones in danger.

The similarity that was found between the two adversities may serve to reconcile the debate concerning the dominance of fear versus hope. It has been claimed that fear would generally dominate hope in individuals’ lives because fear is a primary emotion, while hope is a secondary emotion, and that only under special circumstances individuals holding great determination may replace fear dominance with hope domination [56]. Our data do not support this view, showing that the general Israeli public responses do not follow this fear dominance theory. Rather, in coping with the two different present adversities, hope generally constitutes a stronger determinant of coping compared to fear. Thus, the present results show that, as expected, hope impacts coping with adversity better than fears of potential and actual relevant risks. Furthermore, hope probably impacts the coping responses in the context of the COVID-19 pandemic more substantially than in a limited armed conflict. This can be seen in the lowest values of the hope paths of the two distress-predicted variables (anxiety and depression) in the armed conflict sample compared with the COVID-19 sample. This result may reflect the fact that this pandemic is characterized by a continuous unpredictability and lack of certainty, whereas the Israeli public is quite used to limited armed conflicts, whose nature and outcomes are well known. In addition, as can be expected, the coping of the public in a security situation is affected more readily by the security fears, while coping with the long-lasting pandemic is predicted to a greater extent by economic fears. An important implication of this finding is the need of governments to establish regulations that aim to minimize the economic threat during the pandemic, in order to raise the hope of the population for a better future once the pandemic is contained.

Despite these results, evidently no single factor constitutes a predictor of coping with stress. This is mostly true for anxiety and depression predictors, which are strongly related to worries concerning real and imaginary threats. Worry was defined as “the uncontrollable negative cognitive activity associated with anxiety” [69] (p. 12). This linkage of worries and anxiety has been previously emphasized [70,71]. It has been claimed that worries are an integral component of anxiety disorders, associated also with depression [72]. More specifically, an individual’s level of anxiety and depression during the COVID-19 pandemic was found to be associated with the number of worries experienced during stressful conditions [73].

### Limitations

A limitation of this study is its sampling method. Though using an internet panel company to collect the data verifies a representative sample (from a demographic point of view), it should be noted that only those willing to take part in the survey are included. If these individuals are somewhat different from those who refuse to take part in the survey, it may also impact its representativeness. Another limitation is that, due to budget constraints (the survey was distributed in Hebrew only), the study was conducted only among the Israeli Jews population (the majority). Finally, the study is based on subjective self-reports of the general public in Israel and, thus, may depend on the respondents’ awareness and willingness to give accurate responses. This subjective data collection method always raises the question of whether the data accurately represent the psychological responses.

## 5. Conclusions

Our findings and assertions concerning the advantage of hope over fear in coping with stress is supported by Snyder, who claimed that hope should be regarded as a key protective factor for benefits of high hope in psychological development [74]. Hope has strong positive associations with a variety of psychosocial processes and outcomes, such as emotional adjustment, positive affect, life satisfaction, sense of purpose, quality of life, and social support [75]. Another study provided preliminary evidence that hope, as a strength, can buffer against the effects of acute negative life events [76]; another study confirmed the benefits of high hope level on individuals’ academic achievement, behavioral development, and personal adjustment [77].

The present study adds to the research on coping with stress of adversities, because, to the best of our knowledge, it is the first study exploring the relative impact of level of hope, as compared to fear of threats, as predictors of indicators of coping with two distinct mishaps. Three major conclusions can be drawn: a. Hope is a better and more consistent predictor of both coping and coping suppressing expressions in face of adversity. b. The advantage of hope over fear as a predictor of coping is replicated in two different stressful conditions (global pandemic and armed conflict). c. Fears of specific threats indeed predict coping under appropriate circumstances.

In conclusion, as far as these two investigated crises are concerned, hope is a stronger and more consistent predictor of coping compared with fears raised by actual or potential threats (in the current study: health, security, economic, and political threats). Our results support the argument that many people hold a basic level of hope helping them overcome both minor and major stresses. Hope helps people face diverse stressful events and prevents yielding to the fears they generate. We believe that the advantage of hope over fear in predicting coping reactions is not limited to the current investigated adversities. Additionally, our findings may suggest a central practical implication concerning the importance of emphasizing sources of hope over sources of fear in communicating with people at times of adversities.

### Future Research

The present study results pertain to the impacts of hope and fears of environmental threats on psychological coping with two different adversities: the COVID-19 pandemic and an armed Israeli–Palestinian conflict. There is reason to believe that the significant and consistent predictions of indicators of coping by hope, which are replicated in both of the harsh conditions, may be generalized to other dangerous events. Hope is an unfailing predictor of psychological coping [13]. It may appear, however, that the somewhat lower impact of hope on coping with an armed conflict, compared to the epidemic, may be a specific characteristic of the conflict faced by the Israeli public. The impacts of different fears on coping with these two adversities are likely to vary according to the specific conditions of different populations. Thus, we suggest that similar research comparing two different adversities in other populations is needed to confirm the current results.

The present results were obtained using representative samples of the Israeli population. Despite the replication of the results in response to two different dangers, additional studies, with other populations and/or other adversities, are required to affirm the role of hope in coping with stress. Specifically, a replication should reexamine the role of hope in less extreme daily stresses encountered by people, as well as in chronic cases of stress to assess its generality. The results also indicate that, under certain conditions, different measures of coping suppression are also predicted by different fears [78]. Further research should examine what conditions affect each of these responses, and in what way.

## Figures and Tables

**Table 1:** Demographic characteristics of the participants.

		COVID-19 (*N* = 699)	Armed Conflict (*N* = 647)
Variable	Values	*n* (%)	*n* (%)
Age groups	18–30	128 (18)	96 (15)
	31–40	170 (24)	145 (22)
	41–50	137 (20)	136 (21)
	51–60	123 (18)	116 (18)
	≥61	141 (20)	152 (24)
Gender	Women	330 (47)	297 (46)
	Men	369 (53)	350 (54)
Income level	Below average	394 (56)	339 (52)
	Average	163 (23)	154 (24)
	Above average	142 (20)	154 (24)
Political attitudes	Left	87 (12)	77 (12)
	Center	259 (37)	236 (36)
	Right	353 (50)	334 (52)
Religiosity	Secular	351 (50)	328 (51)
	Traditional	196 (28)	177 (27)
	Religious	97 (14)	91 (14)
	Orthodox	55 (8)	51 (8)

**Table 2:** Standardized estimated of path analyses of four type of threats and hope predicting individual and societal resilience, well-being, anxiety and depressive symptoms during the COVID-19 pandemic (*n* = 699) and armed conflict (*n* = 647).

Predictor	Predicted	COVID-19 Estimate	Armed Conflict Estimate
Hope	Individual resilience	0.29 ***	0.36 ***
	Societal resilience	0.47 ***	0.50 ***
	Well-being	0.37 ***	0.41 ***
	Anxiety	−0.19 ***	−0.07 *
	Depression	−0.30 ***	−0.23 ***
Political threat	Individual resilience	0.13 ***	0.15 ***
	Societal resilience	−0.20 ***	−0.17 ***
	Well-being	0.04	0.07 *
	Anxiety	0.04	−0.01
	Depression	0.02	−0.01
Economic Threat	Individual resilience	−0.13 ***	−0.02
	Societal resilience	−0.07 *	−0.06
	Well-being	−0.31 ***	−0.19 ***
	Anxiety	0.23 ***	0.13 ***
	Depression	0.28 ***	0.24 ***
Health Threat	Individual resilience	−0.17 ***	−0.24 ***
	Societal resilience	0.12 **	0.01
	Well-being	−0.15 ***	−0.28 ***
	Anxiety	0.27 ***	0.17 ***
	Depression	0.15 ***	0.16 ***
Security Threat	Individual resilience	−0.12 **	−0.19 ***
	Societal resilience	0.04	0.05
	Well-being	−0.07 *	−0.05
	Anxiety	0.11 **	0.41 ***
	Depression	0.13 ***	0.21 ***
R^2^ (explained variance)	Individual resilience	0.20	0.31
	Societal resilience	0.30	0.33
	Well-being	0.38	0.45
	Anxiety	0.32	0.36
	Depression	0.35	0.36

* *p* < 0.05; ** *p* < 0.01; *** *p* < 0.001.

## Data Availability

All data accumulated in the study are available to the authors. Data are not published openly due to privacy issues, but analyzed data are available from the authors upon request.
